# Measuring Biosignals with Single Circuit Boards

**DOI:** 10.3390/bioengineering9020084

**Published:** 2022-02-21

**Authors:** Guido Ehrmann, Tomasz Blachowicz, Sarah Vanessa Homburg, Andrea Ehrmann

**Affiliations:** 1 Virtual Institute of Applied Research on Advanced Materials (VIARAM); 2 Institute of Physics—Center for Science and Education, Silesian University of Technology, 44-100 Gliwice, Poland; tomasz.blachowicz@polsl.pl; 3 Faculty of Engineering and Mathematics, Bielefeld University of Applied Sciences, 33619 Bielefeld, Germany; sarah_vanessa.homburg@fh-bielefeld.de (S.V.H.); andrea.ehrmann@fh-bielefeld.de (A.E.)

**Keywords:** ECG, EMG, Arduino, Raspberry Pi, sweat, health condition, health status, elderly, firefighters, sportsman

## Abstract

To measure biosignals constantly, using textile-integrated or even textile-based electrodes and miniaturized electronics, is ideal to provide maximum comfort for patients or athletes during monitoring. While in former times, this was usually solved by integrating specialized electronics into garments, either connected to a handheld computer or including a wireless data transfer option, nowadays increasingly smaller single circuit boards are available, e.g., single-board computers such as Raspberry Pi or microcontrollers such as Arduino, in various shapes and dimensions. This review gives an overview of studies found in the recent scientific literature, reporting measurements of biosignals such as ECG, EMG, sweat and other health-related parameters by single circuit boards, showing new possibilities offered by Arduino, Raspberry Pi etc. in the mobile long-term acquisition of biosignals. The review concentrates on the electronics, not on textile electrodes about which several review papers are available.

## 1. Introduction

Biosignals are measured nowadays for various reasons. In many cases, biosignals such as the ECG (electrocardiogram) or pulse rate, skin temperature or breathing frequency and many other parameters are measured for medical reasons [1,2,3]. Other possible applications are met in many sports disciplines [4,5,6], or even in human-machine interfaces (HMIs), e.g., to control a prosthesis, an exoskeleton, or a robot [7,8,9,10].

As glued electrodes for ECG measurements and rigid systems for other measurements are uncomfortable in long-term usage, many approaches to integrate electrodes into clothes or to prepare textile-based electrodes have been reported during the last decades [11,12,13,14,15,16]. While these attempts result in increasingly reliable soft textile electrodes and sensors for the detection of ECG and many other biosignals, data evaluation still necessitates either rigid electronics or highly specialized flexible electronics, which are not available for all research groups in the textile or medical area [17,18,19]. It should be mentioned that there are also approaches to measure with completely contactless methods [20,21,22,23], which are, however, even more sophisticated and not accessible for all researchers.

Nowadays, a broad variety of single circuit boards is available for this purpose, i.e., microcontrollers or microcomputers, which are often small enough to be integrated in clothes without strongly irritating the wearer if the smaller boards are chosen, or those which are specially designed for textile-integration, such as LilyPad Arduino boards [24,25]. Amongst these single circuit boards, microcontrollers from the Arduino family and Raspberry microcomputers have often been used recently. However, there are diverse other single circuit boards available, such as Espressif microcontrollers, Adafruit Feather, Calliope, Teensy, BeagleBone Black, ATTiny85, or Micro:bit, thus enabling choosing from a broad spectrum of possible single circuit boards for each project [26].

Generally, single circuit boards can either be microcontrollers, like the Arduino family, or microcomputer, such as Raspberry. Typical controllers are ATmega328/P from Atmel, e.g., used in Arduino Uno and Arduino Nano, the ATmega32U4 (Arduino Micro), or the ATtiny85 (Digispark Mini) [27,28]. Operating voltages are usually equal to 5 V (3.3 V in the case of the Arduino Nano 33 BLE), input voltages often equal 7–12 V, with usually very low power consumption in the range of 20 mA. The numbers of digital and analog I/O pins varies. The clock frequency is often equal to 16 MHz (64 MHz for the Arduino Nano 33 BLE). Dimensions are in the range of 53.4 mm × 68.6 mm (Arduino Uno) and larger, but also much smaller, e.g., 12 mm × 26 mm for the Digispark Mini. Similarly, the masses vary between approximately 2 g (Digispark Mini) and 25 g (Arduino Uno). For programming, usually the Arduino IDE is applied.

**Figure 1:**
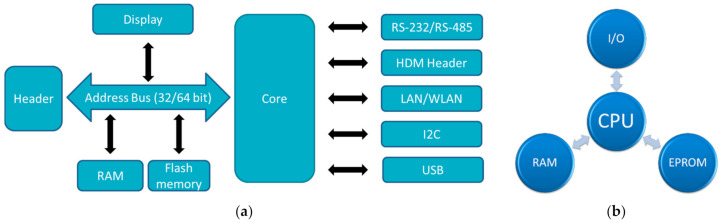
Architecture of (**a**) single board computers; (**b**) microcontrollers. Abbreviations: HDM—High Density Metric; LAN—Local Area Network; WLAN—Wireless LAN; I2C—Inter-Integrated Circuit; RS—Recommended Standard; I/O—Input/Output; CPU—Central Processing Unit; RAM—Random Access Memory; EPROM—Electrically Erasable Programmable Read-Only Memory.

In the Raspberry family, usually chips based on an ARM quad core are applied [29]. The SoC (system-on-chip) in the recent Raspberry Pi 4B is the BCM2711, based on a quad-core Cortex-A72 (ARM v8) with 64 bit and 1.5 GHz, while other Raspberries contain other ARM cores. Most Raspberry Pi contain a quad-core CPU, while the Raspberry Pi Zero/W has only one core and 1 GHz GPU clock frequency. The latter also uses only 512 MB RAM, while the larger Raspberry Pi use 1 GB, or even up to 8 GB memory. This difference is also visible in the dimensions and the mass, with the longest side of Raspberry Pi 4B and Raspberry Pi 3B+ having 85.6 mm, while the Raspberry Pi Zero/W is only 65 mm × 30 mm × 5 mm small, i.e., smaller than an Arduino Uni. It should be mentioned that the Raspberry PI Pico is a microcontroller, not a full microcomputer, and is not much larger than the well-known Arduino Nano. The architectures of common microcontrollers and single-board computers are depicted in Figure 1.

**Figure 2:**
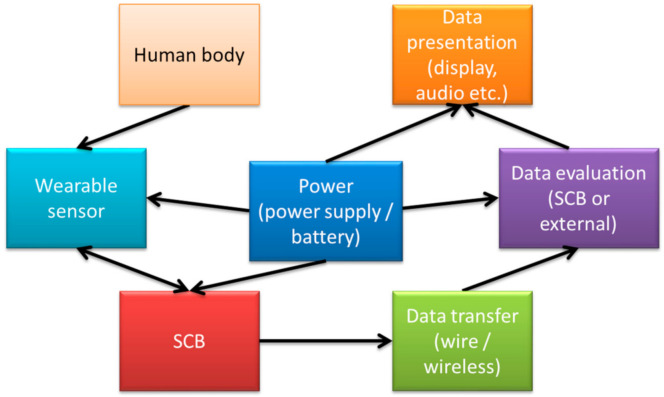
Block diagram of a typical measurement setup using a single circuit board (SCB).

Several single circuit board-based biosignal-detection systems are described in the literature, mostly designed for special applications, such as ECG measurements, while others aim at providing a more general approach or suggest possible applications of such systems for didactic purposes [30]. A general block diagram of the components used in such measurements is depicted in Figure 2.

Da Silva et al., e.g., described a development platform aiming at making physiological measurements available “for everyone” [1]. The group developed the low-cost modular biosignal acquisition hardware platform “BITalino” to enable building biomedical devices in an easier and more reliable way [31,32,33,34,35,36,37]. Other specially designed hardware for biosignal detection include, e.g., the Olimex shield for ECG and EMG measurements, compatible with Arduino like development boards [38,39]. While such specialized hardware platforms usually show a better performance in physiological computing applications, the Arduino and in some cases Raspberry or other microcomputers and microcontrollers are still more popular [40].

This is especially valid since single circuit boards like Arduino or Raspberry are widely available and thus enable creating new healthcare services even in poor regions of the world [41,42]. Many e-health sensor platforms and biosensor-shields are thus based on these single circuit boards, especially on the Arduino, working usually with an ATmega328 chip, or the Raspberry Pi [43,44,45,46,47,48].

Several reviews can be found dealing with biosignal measurements in general [49,50,51,52,53], or with textile integration of single circuit boards [54,55,56], thus these topics are omitted here. This review instead gives an overview of some typical applications of single circuit boards for biosignal detection, mostly based on Arduino or Raspberry Pi, which is recently not available in the scientific literature.

The paper is structured as follows: Starting with the most often applied ECG and pulse measurements, the next sections give an overview of breathing measurements, EMG and EEG including possibilities to control prostheses or robots, bioimpedance, skin temperature, detection of moisture and more in-depth analysis of sweat, followed by a brief overview of other biosignals as well as didactic approaches, using biosignal measurements with inexpensive and easy to handle equipment in school.

This review aims at investigating the possibilities and challenges regarding measuring biosignals with single circuit boards. Papers were collected by searching in the Web of Science and Google Scholar for search phrases like “single circuit board/single board computer/single board microcontroller/Raspberry/Arduino/Digispark” + “biosignal/ECG/pulse/breathing/EMG/EEG/bioimpedance/temperature/moisture/sweat”. It should be mentioned that due to the large number of papers in this broad area, the authors have chosen those that they found important for a general overview, as well as special examples going beyond the most often used techniques. While this choice is necessarily subjective, the authors believe that it is instructive for researchers from other areas, interested in measuring biosignals with single circuit boards, as well as for researchers already working in this area, giving new impulses. 

## 2. ECG and Pulse Measurements

The ECG and, as a simpler measurement with reduced information value, the pulse rate, are among the most important biosignals, enabling evaluation of one of the essential signals derivable from the human body. Similar to a previous study in which most textile-based sensors were found to be related to ECG measurements [14], a majority of biosignal electronics deals with measuring ECG and pulse. 

**Figure 3:**
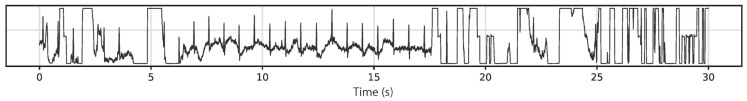
Example signal measured on a steering wheel whilst driving. From [57], originally published under a CC-BY license.

**Figure 4:**
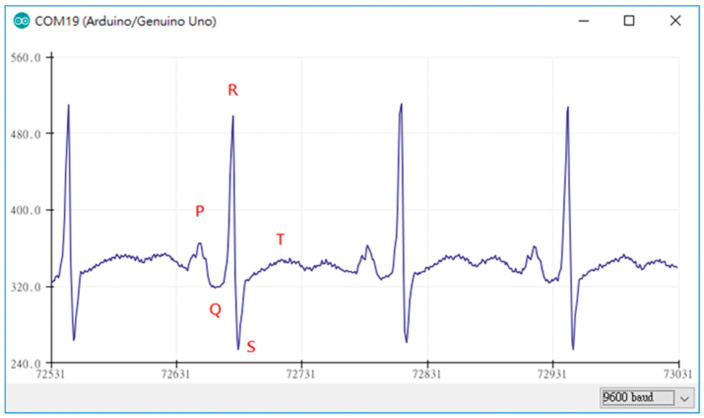
ECG measurement plot obtained by the Arduino device in combination with AD8232. From [66], originally published under a CC-BY license.

Generally, several prerequisites can be defined for ECG measurements. Figure 3 depicts an exemplary measurement taken during using a steering wheel, as it may be useful to monitor a car driver [57]. Here, the main challenges occur when one hand is taken away from the steering wheel, breaking the circuit. However, other potential problems are also visible, such as the typical noise occurring due to insufficient contact between electrodes (especially textile ones) and the skin, as visible in the time range of 7–17 s. Besides, it must be mentioned that in most cases not only the QRS complex, detected when the heart beats, but also the previous P wave and the subsequent T wave (for definitions cf. Figure 4) should be measured to gain all available and medically interesting information [58,59,60].

This means that not only the voltage resolution (the *y*-axis in Figure 3), but also the time resolution (*x*-axis in Figure 3) must be sufficient to detect such signals properly. Typical resolutions are in the range of 500 Hz–10 kHz and 10–12 bits for a voltage range of around 0.5–5 mV, respectively [61,62]. Therefore, the usual 10 bit or 12 bit analog-digital converter (ADC) of Arduino microcontrollers is, for several applications, supplemented by additional ADC modules [63,64]. This section reports examples of how ECG and pulse signals are measured in Arduino and Raspberry based systems.

One of the possible shields or additional hardware parts, added to an Arduino microcontroller, is the aforementioned BITalino. Alves et al. described measuring ECG signals using an Arduino Pro Mini (3.3 V, 8 MHz) connected with a Bluetooth Mate module and a BITalino, to one of whose analog input pins the ECG sensors were connected [32]. During measurements, data from two analog and four digital input pins were written into an array. An application programming interface written in Java controlled the Arduino, setting parameters such as detection mode, baud rate and sampling rate (here 1 kHz). The authors reported on loosing approximately 5 samples per second in test measurements of a synthesized square wave, based on the Arduino’s clock accuracy error of 0.2%, which could be ignored for biometric data evaluation. They showed a comparison of the raw signal with a filtered one, using a low-pass Kaiser filter between 2.5 Hz and 30 Hz, which was well suitable to detect a characteristic ECG signal. 

Ahamed et al. used an Arduino Uno for AD-conversion and signal transmission [65]. They used the internal 10 bit ADC and a sampling rate of 1270 Hz. The signals were transferred to a laptop using a Bluetooth module HC-06 with a baud rate of 38,400 bps. Measurements of ECG were performed on Lead I (between both wrists), using commercially disposable electrodes Bio Protech T716. After filtering the signal by a Butterworth band pass and an elliptic band stop filter of 50 Hz, a flat signal with well visible P and T waves was received.

Lin et al., on the other hand, used an Arduino Mega 2560, based on the ATmega2560 microcontroller, with 54 digital I/O pins and 16 analog inputs to prepare an ECG measurement system [66]. For ECG measurements, the module AD8232 was applied, which is a very small (4 mm × 4 mm) module especially designed for filtering noisy biosignals [67,68]. In this way, smooth ECG signals were monitored, as depicted in Figure 4, and displayed using Matlab [66]. The AD8283 was also used to investigate different electrodes in combination with an Arduino board [69].

Another ECG amplification module, the EKG-V2, was investigated by Branzila and David in combination with an Arduino Uno [70]. The authors reported common output signals of the EKG-V2 module of approx. 200 mV and a maximum current consumption of 2 mA, enabling long-term investigations without steadily exchanging the battery. The Arduino was used to transfer the signals from the ECG amplifier, attached to an analog input, to a laptop where further filtering, signal processing and finally depiction of the signal by a LabVIEW routine was performed. Data transmission from Arduino to a receiver is possible, e.g., using an Arduino BT capable of Bluetooth data transmission [71].

Morales et al. combined pulse rate measurements [72], using a PulseSensor device [73] attached to an analog input of an Arduino Uno, with ECG measurements by applying three electrodes to an Arduino e-Health shield [74]. An additional Wi-Fi shield allowed for sending ECG data to a smartphone or tablet. Further information about the detected ECG signals or a graphical depiction, however, were not given. The PulseSensor was also used in combination with an Arduino Nano [75].

An Arduino Uno was also applied for ECG measurements with active electrodes including an integrated operational amplifier, bipolar capacitively coupled with the human body [76]. Here, an AD8642 and an AD8421 (both from Analog Devices, Wilmington, MA, USA) were tested to measure the bioelectric signals with and without a guard layer of the active electrodes, respectively, and an LTC2338 ADC with 18 bit was used to digitize the analog signals from the front-end. The results showed a significantly improved signal when the measurement equipment was used inside a grounded Faraday cage. Applying active electrodes and an analog front-end ADS1191 (by Texas Instruments, Dallas, Texas, USA), a capacitive ECG measurement was performed on a person sitting on a chair with embedded electrodes. Up to eight bipolar channels were measured with an analog frontend based on the highly integrated chip ADS1299 (Texas Instruments) [77].

In contrast to the previously described project, Abtahi et al. used a Raspberry Pi Model B, based on an ARM processor, instead of an Arduino-based microcontroller [78]. This Raspberry Pi has an Ethernet port, two USB ports, an HDMI port, a 700 MHz CPU and 512 MB RAM, and it supports diverse Linux distributions. As the frontend for a 12-lead ECG measurement, they chose an ADAS1000 (by Analog Devices) with a maximum sample rate of 128 kHz. While the authors were able to measure smooth ECG graphs with this setup, they also mentioned the difficulties to contact the ADAS1000 by manual soldering and that the Raspberry Pi Model B is not compact enough to be integrated into wearable devices. Hafid et al. also used a Raspberry Pi with ADAS1000 to measure a 3-lead ECG [79], while Abtahi et al. suggested using this combination for an affordable part of a homecare system [80].

It should be mentioned that while some research groups have concentrated on developing and testing electronics especially suitable for ECG measurement in combination with Arduino or other single circuit boards, other research groups have gone further and aimed at developing a classification of the measured ECG data, e.g., by artificial intelligence and different neural networks [43,81,82].

Besides the ECG, breathing belongs to the essential biosignals. Breathing measurements based on single circuit boards are thus described in the next section.

## 3. Breathing Measurements

Breathing measurements can be performed in different ways, e.g., by embedding a push switch in a chest belt which counts the breathing rate [83], by measuring the breathing flow near the nostrils by a ventilator [84] or by a thermistor near the nose monitoring the breathing rate [85], but also in much more sophisticated ways, e.g., by measuring a photoplethysmogram, which detects heart rate, arterial blood oxygen saturation and blood pressure, but also allows for detecting respiration [86]. Besides the pure breathing rate, it is also possible to measure CO_2_ content with a so-called capnograph, as suggested by Sing et al., using an Arduino Mega 2560 with an infrared CO_2_ sensor in comparison with a commercial capnography device and finding good agreement between the self-built solution and the commercial one [87].

An interesting application of breathing detection was suggested by Telang who developed a “mouth breathing controller” based on an Arduino Uno, to enable training people to avoid breathing through the mouth [88]. This controller is based on using a proximity sensor to detect an open mouth of a person, making them aware by a noise and a mechanical action that the mouth should be closed, i.e., breathing is not detected. Other applications are more related to the real breathing process. Using an Arduino Nano, Mikha and Aljobouri developed a continuous positive airway pressure device with embedded blower and pressure sensor, controlling the motor speed and pressure set value by a PID controller library, for patients with obstructive sleep apnoea [89].

Breathing detection by temperature measurement was suggested by Patel et al. [90]. They connected a digital temperature sensor TMP102 with a resolution of 0.06 K to an Arduino Due and measured the temperature change near the nostril during respiration, which was found in the range of 2 K and thus well measurable.

A fibre-optical sensor for new born incubator applications was developed by Dhia et al., who used an Arduino Uno in combination with a bent optical fibre used as strain sensor [91]. During breathing, the optical fibres were slightly elongated and thus more strongly bent, resulting in higher losses inside the optical fibre and thus a reduced signal at the detector, attached to the end of the optical fibre. In this way, detecting breathing between 10 and 130 breaths per minute was possible.

An application for asthma patients was suggested by Abinayaa et al. who measured volatile gas concentrations in the environment with a gas sensor, patient activity by a gyro sensor, as well as temperature and humidity, and breathing to enable long-term monitoring of asthma patients [92].

While heartbeat and breathing are the biosignals normally assumed to be most important, other electrical signals can be used to investigate a person’s health, but also to control prostheses, exoskeletons etc. These are described in the next sections.

## 4. EMG Measurements

Electromyography (EMG) signals indicate muscle responses according to stimulation by the brain, transmitted through nerves [93]. To enable patient mobility, EMG measurements should be performed by miniaturized equipment, e.g., based on Arduino microcontroller boards [93]. In this way, recovering patients are supported during training [94]. For patients suffering from myopathy, EMG data can support diagnostics by building up an EMG database, e.g., combining an amplifier AD524 with an Arduino board for real-time AD conversion [95]. On the other hand, a miniaturized EMG sensor, based on the Arduino Pro mini, was used to inform the user about incorrect posture during long-term computer use, in this way reducing neck fatigue [96].

**Figure 5:**
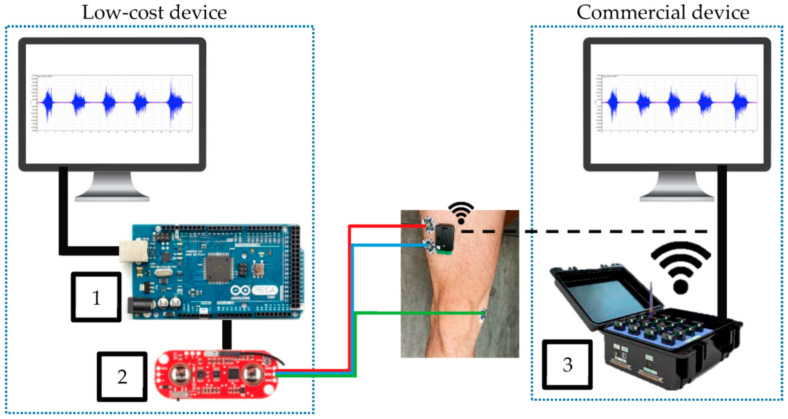
Experimental equipment, comparing Arduino Mega (1) connected to Myoware EMG muscle sensor (2) with a commercial device (3). From [97], originally published under a CC-BY license.

Fuentes del Toro et al. compared a low-cost device, based on an Arduino Mega with a Myoware EMG muscle sensor, with a commercial Delsys Trigno Wireless EMG system (Figure 5) [97]. The authors chose the Arduino Mega to avoid the limitations of memory and power of the smaller boards because data acquisition and filtering required a large memory. Besides the wireless connection of the commercial device, both devices were found to be in good agreement, indicating that a low-cost device based on an Arduino board can be used for this purpose. This result was verified in a study by the same group, investigating muscle fatigue detection [98].

Muqueet nevertheless underlined the very small signals in the range of 50 µV to 1 mV in a broad frequency range of 10 Hz to 3 kHz, making EMG measurements challenging [99]. He used an ESP8266 NodeMCU microcontroller board, which contained a Wi-Fi networking solution and could be controlled by the Arduino IDE, combined with an EMG sensor and surface electrodes, to measure real-time EMG signals and display them by the Serial Plotter of the Arduino IDE.

While the aforementioned studies dealt with EMG measurements for rehabilitation or diagnostic purposes, most projects on this topic aimed at using EMG signals for controlling prostheses, exoskeletons or even robots [10,100,101,102,103,104]. For this purpose, Champaty et al. developed an EMG biopotential amplifier based on the AD620 instrumentation amplifier, connected to an Arduino Uno responsible for signal processing and classification [105]. The gained signals were transmitted to a wheelchair model by a Xbee transceiver. The authors showed that by bending different fingers, it was possible to receive different signal amplitudes, which could be used to control the servo motors in the wheelchair model, in this way enabling easier handling than by the traditional joystick which is not suitable for all patients.

Mundra et al. investigated the accuracy with which pre-defined gestures were identified when an EMG was measured with the MYO armband, connected to an Arduino Uno with HM-10 Bluetooth module [106]. The eight sensors of the MYO armband delivered approximately 8000 values for one gesture. The authors compared quadratic discrimination analysis, K-neighbors classifier, gradient boosting classifier, random forest classifier and others, and found the highest gesture recognition accuracy for the quadratic discriminate analysis and the gradient boosting classifier.

Borisov et al. developed a prototype of an EMG-controlled prosthetic hand, based on an Arduino Mega and the Grove EMG Detector [107]. The prosthetic hand included a feedback system equipped with audio information, visual information, and vibration signals, according to measured signals from a force sensing resistor (FSR), making it easier for the probands to properly grip an object.

**Figure 6:**
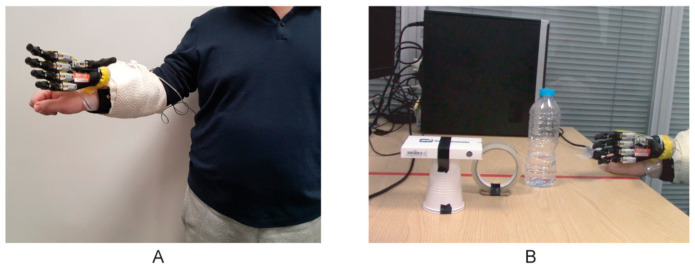
Pick-and-place experiments, (**A**) with a proband wearing the prosthetic hand attached by a special socket, (**B**) showing three objects which had to be grasped and relocated. From [108], originally published under a CC-BY license.

Similarly, Wu et al. suggested an Arduino-based myoelectric control of a prosthetic hand, as depicted in Figure 6 [108]. They decided to use an Arduino, here in combination with a Gravity analog EMG sensor (OYMotion, Shanghai, China) as this board could also be used with other EMG sensors, such as MyoWare (Sparkfun, Niwot, CO, USA) or Grove EMG detector (Seeed Technology Inc., Shenzhen, China). With these systems, users can adjust system settings and learn to control the use of the prosthetic device, based on different control mechanisms. In their experiment, the probands firstly trained defined motions before they should grasp a bottle, a roll tape and a credit card simulator with different possible grips and open the prosthetic hand between these grasps. The authors reported that the trained abstract controller and linear discriminant analysis classifier developed in their study resulted in a faster successful finish to the experiment, than a direct controller. 

Ganesan et al. combined an EMG sensor with an inertial measurement unit (IMU), which is able to detect acceleration, angular velocity and orientation, to develop an upper limb exoskeleton with feedback for rehabilitation [109]. Both sensors were fixed on the good arm to control the exoskeleton around the rehabilitating arm. Before the test, the system was calibrated for each proband by maximum biceps contraction. The system performance was rated between 56% and 82%, depending on the tests and the subjects, indicated that it works in principle, but could be further optimized.

Combining an Arduino Uno with an Olimex shield, working at 256 Hz sampling rate with a 10 bit ADC to detect and filter myoelectric signals, Rahmatillah et al. developed an exoskeleton for both arms to support stroke patients during rehabilitation [110]. The authors suggested applying a Kalman filter to enable missing information from noisy indirect measurements, i.e., here to separate the myoelectric signals from noise and to smooth the signal for a better control of the motor at the rehabilitating arm. In this way, a performance accuracy of 95% could be reached, underlining that such an inexpensive solution can be suitable for active rehabilitation purposes.

Besides EMG signals, EEG (electroencephalogram) signals can also be measured and used for the control of prostheses and other objects, as will be shown in the next section.

## 5. EEG Measurements

EEG signals can be measured, e.g., in basic medical research [111,112], for neurofeedback or biofeedback training, for medical applications, or in many cases be used as an HMI, mostly used to control prostheses, a robotic hand or a wheelchair, based on Arduino or Raspberry Pi [113,114,115,116,117,118]. This is why there is even an OpenEEG project suggesting hardware and software for EEG measurements [119]. Correspondingly, many researchers report on EEG measurements, often based on hardware including single circuit boards.

Saptono et al., e.g., used an Arduino MEGA1280 and programmed a graphical user interface (GUI) EEG analyser [120]. They described the expected signals in the range of 5 µV to 200 µV and the different frequencies between approximately 0.5 Hz and 100 Hz, usually subdivided into the five frequency bands alpha, beta, gamma, delta, and theta. The authors combined an active electrode with different filters and built a GUI based on the BrainBay open-source application, allowing to present alpha and theta wave magnitudes as well as the unfiltered signal and an average frequency.

Pari-Larico et al. used Neurosky’s MindWave, connected by Bluetooth HC-05 to an Arduino Uno board, and an LCD screen as well as LEDs connected to the Arduino Uno to display measured EEG values and levels of attention, respectively [121]. Average attention levels were found to differ between male and female probands (*n* = 46 probands) and partly between different age groups.

Besides these basic measurements, many researchers have concentrated on using EEG measurements as HMI. Mahajan and Bansal, e.g., developed a brain-computer interface (BCI) based on EEG measurements, performed by a neuro headset EMOTIV, which enables detecting a 14-channel EEG [122]. While signal processing was performed by MATLAB on a laptop, an Arduino Uno board was interfaced with MATLAB, enabling controlling the board by the processed EEG signals. The authors used this setup to control an LED by eye blinking, as a proof-of-concept for future applications in home automation and prosthetic control. LED lights were also used as simple actors, representing left and right imaginary hand movements, in a study by Dabas et al. who used a 32-channel EEG to investigate the accuracy given by different classification algorithms based on alpha band signals [123].

Rashid et al. used alpha and beta waves of the EEG for the control of upper limb prostheses [124]. EEG signals were taken by the Emotiv headset with 14 electrodes and a sampling frequency of 128 Hz during defined finger movements. An Arduino Uno was used for filtering and classification of the input data. They concluded that more channels and a better signal-to-noise ratio would be supportive for higher classification accuracy.

An Arduino Uno was also used for the control of a prosthetic hand by an Emotiv EEG headset, as reported by Abu Kasim et al. [125]. A GUI was again based on LabVIEW, and LabVIEW and Arduino were connected by VISA virtual instruments. The face expressions “look right” and “smile” were used to control “hand open” and “hand close” of the prosthetic hand, respectively. The authors reported on complicated signal processing due to interference with muscle movement, cardiac signals, and eye blinks, but generally found this approach to be suitable to control a prosthetic hand by EEG signals.

**Figure 7:**
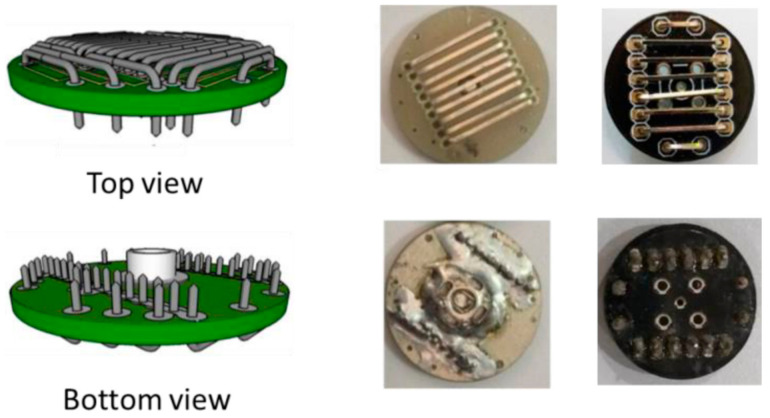
Electrode design (**left** panel) and implementation (**right** panel). From [126], originally published under a CC-BY license.

Instead of applying a commercial EEG electrode system, Pratama et al. prepared dry-active electrodes with pre-amplifier modules INA118 on their own, as depicted in Figure 7 [126]. In this way, they could produce a low-cost system from easily available components. Noise of the pre-amplifier was reduced by a driven-right leg circuit from ModularEEG design, resulting especially in common-mode voltage and power-line interference reduction. Butterworth filters were used to define the desired frequency range. An Arduino Uno was used with the built-in 10-bit ADC to reduce system complexity. Data transfer by Bluetooth HC-06 was chosen due to low power consumption, low price, and ease of connection. Besides, an SD card module was included. With this equipment, it was possible to represent the higher EEG frequencies above 10 Hz properly, while the authors explained that smaller electrodes and some additional electronics would further support the measurements.

While EMG and EEG measurements are often performed to control prostheses, wheelchairs, robots, etc., a health-related measure is the bioimpedance allowing evaluating water, fat, muscle contents and other parameters of the human body. 

## 6. Bioimpedance Measurements

While many body-related physical parameters are related to direct current or typically measured by direct-current instruments, the bioimpedance is an alternating current resistance, i.e., it consists of a real part (resistance R) and an imaginary part (reactance X), which can also be displayed as a vector with a magnitude Z and an angle which defines the deviation of the vector from the direction of the real part [127]. While different commercial measurement instruments are available, which either give the bioimpedance at a standard frequency of 50 kHz or allow for measuring bioimpedance spectroscopy, e.g., in the range of 5–500 kHz, measurements on biological tissues are always error prone. This leads to the situation that even commercial instruments are not always comparable, and there is no full agreement about the correct equivalent circuit diagram for the human body [128]. Ain et al. compared nine different equivalent models by means of a simulation [129]. With new electrical components entering the market, many researchers have become aware of the new possibilities to create their own low-cost bioimpedance analysis (BIA) [130,131] or bioimpedance spectroscopy (BIS) devices [132], often based on Arduino, Raspberry Pi, or other single circuit boards.

In a recent study, Ain et al. developed an Arduino-based bioimpedance spectrometer with an AD9850 programmable function generator, an AD620A instrument amplifier, an alternating-to-direct current converter AD536A and a POA2134 OpAm of the voltage-controlled current source [133]. An Arduino Nano controlled the frequency of the AD9850 module by serial data between 0 Hz and 40 MHz. The device was tested for several probands and found to give highly stable current results up to a frequency of 200 kHz and reliable results for frequencies up to 100 kHz. With these components, the group found an error below 10% for frequencies below 110 kHz [134]. Previously, they used the same components to detect the fat level of a human body by bioimpedance measurements and found a very good correlation with the measurements performed with a commercial body fat device [135].

An often used integrated circuit (IC) is the impedance analyser AD5933 (Analog Devices) with the corresponding evaluation boards. Apátiga et al. compared scientific literature about the EVAL-AD5933EBZ with the PmodIA, both evaluation boards based on the AD5933, in combination with Arduino and Raspberry as well as with other microcontrollers [136]. They underlined the importance of using a microprocessor, which can apply an external clock signal to the integrated circuit or evaluation boards to expand their bandwidth, and mentioned the missing I2C protocols for the communication between AD5933 or evaluation boards and the controlling microprocessor for the investigated studies.

With the same IC, Harvey and Mendelson developed a portable sensor, based on an Arduino Nano [137]. They reported measuring impedance values between 180 Ω and 165 kΩ. Accuracies for well-known electrical circuits, combining ohmic resistances and capacitors, were found to be in the range of a few percent of Z and a few degrees, respectively, while measurements with silver-coated electrodes on a proband’s forearm were found to be highly repeatable.

Ching and Chen built a 2D imaging system based on bioimpedance measurements with other modules, i.e., a function generator MAX038, a Howland current source circuit generating a constant current, an instrumentation amplifier AD620, connected to an Arduino Uno [138]. A multiplexer circuit built from analog multiplexers CD4051 was used to open and close channels in a measuring unit, taking measurements at different positions which contacted the skin by four electrodes per position, allowing for four-wire measurements at (8 × 8) positions. The system was found to give reliable results in the frequency range of 10 Hz to 50 kHz and was suggested for telemedicine applications.

Besides these highly sophisticated bioimpedance measurements, several other parameters exist that can more easily be investigated using single circuit boards.

## 7. Skin Temperature Measurement

**Figure 8:**
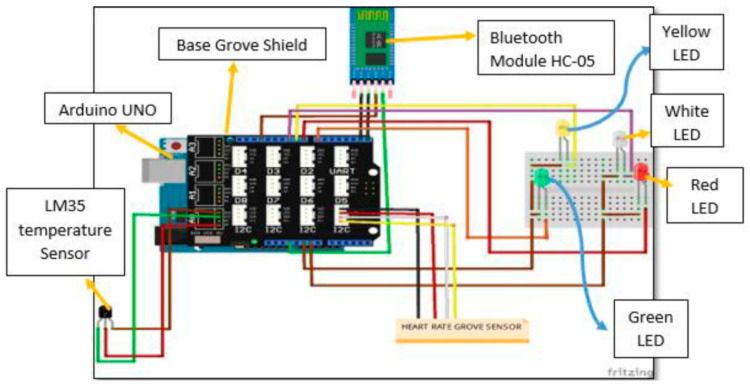
Circuit diagram of the temperature and heart rate monitoring system. From [144], originally published under a CC-BY license.

Temperature can, in the easiest way, be measured by the resistance change in a conductive wire or by a thermocouple consisting of two wires from different metals. It can be measured solely [139,140,141], but it is often combined with heart-rate measurements and suggested for critical situations where patients do not have access to a doctor or hospital [142,143,144,145]. Such a dual-measurement setup is depicted in Figure 8 [144]. Here, the Grove-Heart rate sensor for the fingertip and the LM35 temperature sensor work as inputs for the Arduino Uno, a Bluetooth module HC-05, transmitting signals to a laptop, and LEDs to display warning messages or abnormal heart rates. The authors reported measuring reliable heart rates and skin temperatures. Comparison with a conventional thermometer and a conventional digital pulse monitor, respectively, showed mostly identical readings and only few outliers for short durations.

A comparison between the temperature sensors LM-35 (contact measurement) and MLX-90614 (contactless measurement) was performed by Rahimoon et al. who used an Arduino CT-UNO controller with a Wi-Fi shield [146]. The authors found clear differences between both sensors, which may be attributed to the typical problem of pyrometers (i.e., radiation thermometers) that unknown emission ratios of objects under investigation can result in significant deviations between measured and real temperature.

Besides medical applications, skin temperature measurements can be used for other purposes. Perkasa et al., e.g., used infrared temperature measurements to detect the presence of a person and to switch on the light in this case [147]. The sensor used was a passive infrared receiver (PIR), coupled to an Arduino Uno. Alcoran Alvarez et al. used non-contact body temperature monitoring in combination with an ultrasonic distance sensor with an Arduino Uno for automated social distancing, giving an alarm when the required distance was below the defined limit and the body temperature of the person opposite it was above the normal value on an adult’s forehead [148].

## 8. Moisture Detection

Moisture on the skin can significantly modify the contact resistance between an electrode and the skin, making this parameter highly important for many of the aforementioned measurements. Nevertheless, only few attempts to measure skin moisture by single circuit boards were found in the literature. Sinha et al. used this value, measured by the skin resistance, in combination with heart rate measurements to create a polygraph, based on an Arduino Uno [149]. Similarly, Apostolidis and Tsiatsos used resistance measurements between hands and feet by an Arduino board to evaluate student emotions during learning [150].

Yang et al. used a digital humidity and temperature sensor SH15 (Sensirion AG, Stäfa, Switzerland) with a capacitive humidity sensor and a semiconductor temperature sensor, attached to an Arduino Uno, to measure both values in wheelchair cushion to prevent patients from pressure ulcers [151]. Using a humidity sensor to detect wet diapers was suggested by Rahman et al. who printed conductive lines on the diaper surface, connected them by pressing studs to an Arduino board and used the Arduino serial monitor to evaluate the state of humidity [152]. For smart wound hydration monitoring, Sattar et al. applied an Arduino Uno with MAX30100 heart rate sensor and LM35 temperature sensor to measure wound hydration indirectly, correlating these biomarkers with the wound hydration by a fuzzy inference system [153].

Going one step further, sweat may not only be monitored as a fluid, but also examined further with respect to its contents. These measurements are described in the next section.

## 9. Sweat Analysis

Investigating the glucose level of diabetic patients are among the measurements that have to be performed several times per day by severely ill patients. One possibility to measure this parameter in a non-invasive way was suggested by Nivetha et al. who measured the salt content in sweat instead, which was correlated with the glucose level [154]. They connected copper electrodes to an Arduino Uno, measured both values for healthy and diabetic probands and could verify the correlation between them.

The pH value of the sweat was measured in a microfluidic device including a pH colour indicator in which Lilypad Arduino microcontrollers were used to control micro-LEDs acting as a light source and light detector, respectively [155]. A similar approach was reported by Curto et al. who used a textile-based micro-fluidic platform including a pH sensitive dye, combined with an LED as light source and a photo sensor as detector [156].

A Bluetooth modem sent the received data from the Arduino to a laptop. In this way, pH ranges typically found in the human sweat during exercises could be measured. Wu et al. developed a flexible sensor to measure the pH value of sweat, based on an antimony electrode as ion selective electrode, enabling pH measurement by measuring the output voltage (in the range of some hundreds of millivolts) of the pH sensor [157]. On the other hand, Sood et al. developed a watch measuring the pH value of the sweat by measuring the hydrogen ion concentration to evaluate the glucose level of diabetic patients non-invasively [158].

The next section discusses some other biosignal measurements which were less often found in the literature.

## 10. Other Biosignals

Besides the aforementioned biosignals, which are often measured by single circuit boards, either because these measurements are relatively simple, or because they are important for medical or other purposes, there are some other possibilities that are less often mentioned. 

Arami et al., e.g., investigated the possibility to use Hall elements to measure knee flexion-extension in a smart knee prosthesis [159]. Blood pressure was continuously measured by Kuncoro et al., using an LED/photodetector system as a photoplethysmograph and an Arduino Beetle board for evaluation of the optical data, giving pulse and blood pressure [160].

Stress measuring was performed by detecting the skin resistance [161] or by using multiple biosignals, including EEG, ECG, EMG, and skin resistance [162]. Combining pressure and acceleration sensors, D’Addio et al. developed a sensory sock, connected to a LilyPad Arduino [163]. As a method to control human-machine interaction, Martínez-Cerveró et al. suggested electro-oculography (EOG) signals, evaluated by a Raspberry Pi [164].

Finally, besides the technical component, biosignals can also be measured due to didactical reasons.

## 11. Didactical Approaches

Several researchers have reported on using biosignal measurements with low-cost hardware, often based on single circuit boards, as a possibility to motivate students. While these approaches were not directly related to high-level research, they may nevertheless be regarded as a base to estimate in which direction single circuit board measurements can progress, aiming at making easier and more reliable biosignal measurements possible without using much more sophisticated or highly expensive specialized additional equipment. 

Warren and DeVault described such an approach for a cross-course senior design project [165]. Polo et al. developed a didactic prototype for ECG, EEG, EMG and EOG signal measurements [166]. Other toolkits, based on low-cost hardware and software, were developed by da Silva et al. [33], Abtahi et al. [78], Puente et al. [167] and others [168,169,170,171].

These reports indicated the opportunity to motivate scholars and students of all ages to learn more about physics and chemistry, electronics and programming by linking these topics to biosignals.

## 12. Discussion

As this review shows, many possibilities on the one hand and challenges on the other hand, are reported in the recent scientific literature regarding biosignal measurements with single circuit boards. A brief overview for the here described possible applications is given in Table 1. In all categories, Arduino boards were most often used. 

The main advantage in all cases was the mobility enabled by small single circuit boards, offering the possibility to perform long-term measurements on patients without heavy disturbance of their comfort. Besides, the low costs in comparison to highly sophisticated data acquisition systems must be mentioned, as well as the ease of use. These points are especially important regarding long-term medical measurements and digitalization of medical treatment, but also regarding the possibility to build inexpensive myoelectric prostheses, making them more affordable in low-income countries. 

The challenges, on the other hand, are mostly related to the relatively low computational power and memory of the recent single circuit boards, as compared to recent laptops and other personal computers. Besides, there are many challenges that are identical with those occurring when laptops or similar computers are used, related to producing and positioning electrodes, evaluating the measured signals and interpreting them reliably in terms of possible medical issue. Due to the fast developments during recent years, it can be expected that the technical problems will in a few years be solved, making single circuit boards highly interesting for biosensing applications. It can be hoped that this will at the same time lead to more research groups concentrating on the—then affordable—biosignal measurements and thus better solutions for the still existing problems of reliable textile and other long-term electrodes.

## 13. Conclusions

With single circuit boards becoming more and more powerful, they are increasingly being used to measure various biosignals. This review gives a brief overview of the broad bandwidth of possible signals, detected by such low-cost boards and in most cases combined with low-cost sensors and software.

Typical research and development areas in which such single circuit boards are used are ECG and pulse measurements, EEG and EMG, with both of the latter often applied to control prostheses, exoskeletons or even robots. Other medically important subjects are bioimpedance and breathing measurements, skin temperature and moisture detection as well as sweat analysis. Besides these technical reasons, measuring biosignals by inexpensive and easy to handle single circuit boards is also reported as a good didactical approach, to make students familiar with sensors and programming.

While challenges remain, usually correlated to the relatively low-level AD converters used on most of these boards, limited measurement frequencies and memory, the promising studies reported in the last few years suggest that further development of the different single circuit boards will further increase the possible applications and enable new mobile, low-cost systems with high reliability and reproducibility for biosignal measurements.

## Figures and Tables

**Table 1:** Possibilities and challenges of measuring different biosignals with single circuit boards.

Biosignal	Possibilities	Challenges
ECG and pulse	Mobile long-term measurements possible	Filtering may necessitate too much computation power, i.e., an additional laptop
Breathing	Mobile measurements of volatile patients, e.g., with asthma	Often special sensors near the mouth necessary for a reliable measurement
EMG	Mobile EMG measurements for myopathy patients, posture correction and controlling soft robots/prostheses etc.	Limitations of memory and power of Arduino Uno and other small boards
EEG	Combination with commercial EEG electrode systems possible Enables controlling prosthetic hand etc.	Complicated sensors and sensor positioning Complicated interpretation of data
Bioimpedance	Low-cost bioimpedance spectroscopy gives more information than common 50 kHz measurement	Difficult measurement setup due to high skin resistance and AC measurement Difficult interpretation of the results
Skin temperature	Broad variety of sensors available, based on different physical principles	Skin contact must be ensured
Moisture	Often simple sensors and measurement	More complicated sensor for wound fluid detection necessary
Sweat analysis	Non-invasive glucose level detection of diabetic patients	Sometimes laptop needed in addition
Didactical approaches	Raising students’ interest Toolkits available	None reported

## Data Availability

In this review paper, no new data were created.
